# TNF-R1, an immune checkpoint in melanoma?

**DOI:** 10.18632/genesandcancer.81

**Published:** 2015-09

**Authors:** Florie Bertrand, Céline Colacios, Bruno Ségui

**Affiliations:** INSERM UMR 1037, CRCT, Toulouse, France

**Keywords:** TNF, CD8+ T lymphocytes, apoptosis, tumor-infiltrating lymphocytes

Through binding to and activation of two receptors, TNF-R1 and TNF-R2, Tumor Necrosis Factor α (TNF) modulates various biological processes in the immune system. A growing body of evidence in the literature indicates that TNF may behave as a tumorigenic cytokine, facilitating cancer cell immune escape and tumor progression. We have recently provided evidence that host TNF signaling impairs CD8+ T cell-dependent immune response against melanoma. TNF blockade (with Etanercept) or deficiency (TNF knock out) facilitates the accumulation of CD8+ Tumor-Infiltrating Lymphocytes (TILs), thereby limiting the growth of melanoma cell lines, which express Major Histocompatibility Class 1 molecules (MHCI) at high levels. Similar findings were observed in mice lacking TNF-R1, but not TNF-R2, indicating that host TNF-R1 plays a critical role in limiting the establishment of such a CD8+ T cell-dependent immune response against melanoma under our experimental conditions [1].

We and others have documented that the factor associated with neutral sphingomyelinase activation (FAN), which is an adaptor protein of the TNF-R1 [2], enhances TNF-induced apoptosis [3], leukocyte migration [4] and antibody immune response towards a thymo-dependent antigen [5]. In addition, downregulation of FAN in B16 melanoma cells decreases TNF-induced B16 melanoma motility and invasion [6]. We recently investigated the consequences of host FAN deficiency on the tumor growth of B16 melanoma cells. In sharp contrast to what we observed in mice lacking either TNF or TNF-R1, the tumor growth of B16K1 cells was not impaired in FAN-deficient mice as compared to their wild-type counterparts (Montfort and Ségui, unpublished data). The latter observation suggests that the host TNF-R1 signaling, which likely represents an immune checkpoint facilitating B16K1 melanoma growth, does not critically involve the FAN-dependent neutral sphingomyelinase activation. The role of FAN may thus be restricted to some, but not all, TNF-dependent immune responses. Accordingly, the TNF-dependent anti-infectious immune response towards *Listeria monocytogenes* is not compromised in mice lacking FAN [5].

**Figure 1:**
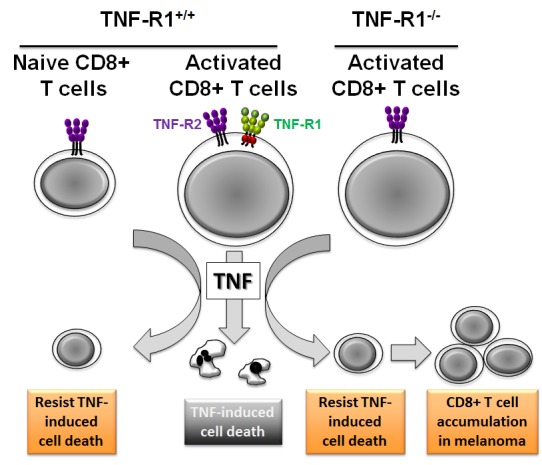
TNF triggers TNF-R1-dependent cell death of activated CD8+ T cells, limiting their tumor accumulation in melanoma In contrast to naive CD8+ T cells, activated CD8+ T cells express TNF-R1, which transduces cell death in response to TNF and likely constitutes an immune checkpoint in melanoma. TNF-R1 deficiency impairs TNF-induced CD8+ T cell death. Inhibiting TNF or TNF-R1 may enhance CD8+ T cell tumor infiltration, thereby limiting melanoma progression.

One of the main mechanisms that likely accounts for the TNF-dependent immunosuppression in our experimental mouse melanoma model is the capacity of TNF to trigger cell death of activated CD8+ T cells. Whereas TNF potently induced cell death of activated CD8+ T cells as documented by others, it remained unclear which TNF receptor is required for this process. On the one hand, activated CD8+ T cells expressed TNF-R2, which mediated activation-induced cell death [7]. On the other hand, exogenous TNF elicited cell death in concanavalin-A activated CD8+ T cells in a TNF-R1-dependent manner [8]. Of note, the latter study was carried out in the presence of cycloheximide to artificially sensitize lymphocytes to TNF. The use of cycloheximide may be a source of artefact and is unlikely appropriate to evaluate the physiological role of TNF and its receptors in CD8+ T cell death. Under our experimental conditions in the absence of cycloheximide, TNF-induced cell death of activated CD8+ T cells occurred mainly through a TNF-R1-dependent process [1]. This conclusion is supported by the following findings: (i) naive CD8+ T cells, which expressed TNF-R2 but not TNF-R1, resisted TNF-induced cell death; (ii) activated CD8+ T cells, which significantly expressed both TNF-R1 and TNF-R2, were sensitive to exogenous TNF; (iii) TNF-R1-deficient CD8+ T cells were fully resistant to TNF-induced cell death; (iv) TNF-R2 deficiency minimally affected CD8+ T cell death in response to TNF. The role of TNF-R1 as an immune checkpoint in melanoma gets further credence as illustrated by our data showing that the accumulation of activated CD8+ T cells into melanoma was facilitated by TNF-R1 deficiency in an adoptive transfer experiment performed in CD8-deficient hosts [1] (Figure 1).

Our recent findings demonstrate that TNF-R1-dependent TNF signaling impairs CD8+ TIL content and likely constitutes a potent immune checkpoint in melanoma, facilitating melanoma progression. Targeting either TNF or TNF-R1 may reduce the death of activated CD8+ TILs in patients affected with melanoma and thus may constitute an emerging immunotherapy to enhance CD8+ T cell-dependent immune response against melanoma. Clinical trials are necessary to evaluate the therapeutic value of TNF blockade strategy in melanoma patients and define the eligibility criteria such as (i) TNF expression in melanoma biopsies, (ii) pre-existing CD8+ T cell infiltration, (iii) MHCI expression by melanoma cells.
